# 

**Published:** 1892-01

**Authors:** 


### CONTENTS.

COMMUNICATIONS.

The Thirty-sixth Annual Meeting of the Michigan Dental Association.......... 1
Over Work.......... 31

PROCEEDINGS.

Twenty-fifth Annual Meeting of the Ohio State Dental Society.......... 35

CORRESPONDENCE.

The Electro-Deposit Plates.......... 39

EDITORIAL.

Aluminum—The Metal of the Future.......... 41
The World's Columbian Exposition.......... 45

